# The evolution of dermal shield vascularization in Testudinata and Pseudosuchia: phylogenetic constraints versus ecophysiological adaptations

**DOI:** 10.1098/rstb.2019.0132

**Published:** 2020-01-13

**Authors:** François Clarac, Torsten M. Scheyer, Julia B. Desojo, Ignacio A. Cerda, Sophie Sanchez

**Affiliations:** 1 Department of Organismal Biology, Subdepartment of Evolution and Development, Uppsala University, Norbyvägen 18A, 752 36 Uppsala, Sweden; 2 Paleontological Institute and Museum, University of Zurich, Karl Schmid-Strasse 4, 8006 Zurich, Switzerland; 3 CONICET, División Paleontología Vertebrados, Museo de La Plata, Paseo del Bosque s/n°, B1900FWA La Plata, Argentina; 4 CONICET, Argentina y Instituto de Investigacion en Paleobiología y Geología, Universidad Nacional de Río Negro, Museo Carlos Ameghino, Belgrano 1700, Paraje Pichi Ruca (predio Marabunta), 8300 Cipolletti, Río Negro, Argentina; 5 European Synchrotron Radiation Facility, 71 Avenue des Martyrs, CS-40220, 38043 Grenoble Cedex, France

**Keywords:** acidosis buffering, cutaneous respiration, heat transfer, historical constraints

## Abstract

Studies on living turtles have demonstrated that shells are involved in the resistance to hypoxia during apnea via bone acidosis buffering; a process which is complemented with cutaneous respiration, transpharyngeal and cloacal gas exchanges in the soft-shell turtles. Bone acidosis buffering during apnea has also been identified in crocodylian osteoderms, which are also known to employ heat transfer when basking. Although diverse, many of these functions rely on one common trait: the vascularization of the dermal shield. Here, we test whether the above ecophysiological functions played an adaptive role in the evolutionary transitions between land and aquatic environments in both Pseudosuchia and Testudinata. To do so, we measured the bone porosity as a proxy for vascular density in a set of dermal plates before performing phylogenetic comparative analyses. For both lineages, the dermal plate porosity obviously varies depending on the animal lifestyle, but these variations prove to be highly driven by phylogenetic relationships. We argue that the complexity of multi-functional roles of the post-cranial dermal skeleton in both Pseudosuchia and Testudinata probably is the reason for a lack of obvious physiological signal, and we discuss the role of the dermal shield vascularization in the evolution of these groups.

This article is part of the theme issue ‘Vertebrate palaeophysiology’.

## 1. Introduction

The vertebrate post-cranial dermal skeleton is composed of bony scutes which ossify within the dermis [1–4]. The presence of these bony elements varies taxonomically, and the resulting shield morphology results from both the shape and the relative position of the dermal plates. These bones can be juxtaposed or articulated as observed in stem archosaurs [5,6], in pseudosuchians [7–9] and some squamates [10]; they can also be fused as in turtles [11,12] and xenarthrans [13,14] or be isolated as in some ornithischian and sauropod dinosaurs [15–18].

Continuous shields of osteoderms (e.g. Aetosauria, Xenarthra) or bony scutes (e.g. Testudinata) have mostly been considered for their protective aspects against predators [19–23]. However, experimental investigations on turtles have shown that their dermal shield would also have ecophysiological functions. Indeed, the bone tissues composing the shield would be able to buffer the acidosis which is caused by blood pH decrease after both the blood CO_2_ pressure has increased and the lactic acid (lactate) has been produced via fermentation during prolonged apnea [24–28]. Bone acidosis buffering consists of supplying mineral elements such as (1) bicarbonates that can bind to the free protons which are dissolved in the blood plasma (due to respiratory acidosis) and (2) calcium that can complex with the lactate and thus inhibits its acidity (in answer to metabolic acidosis) [29].

Such a physiological process has also been identified in the osteoderms of crocodylians [30] which are known to be semi-aquatic animals, derived from terrestrial ancestors [5,8,31]. In addition, crocodylian osteoderms are also involved in heat transfer with the environment during emerged and semi-emerged basking periods [17,23,32] via the enclosed vessels of which blood flow is controlled by cardiac activity and vasomotion, thus regulating the distribution of heat to the vital organs [33–35]. Even though no specific studies have yet been performed on this aspect in the testudinatans, their dermal shield must also be involved in heat transfer [36], since it covers the majority of the body surface while enclosing peripheral blood vessels within bone cavities [37,38].

We quantified the post-cranial dermal bone vascular area as a proxy to assess the number and size of the blood vessels that are both enclosed within bony cavities and closely in contact with the apical bone surface when a superficial ornamentation is present. Indeed, the sculptural elements that compose the bone ornamentation are known to provide vascular openings contributing to the dermal plate global vascularization by conducting blood vessels to the overlying soft dermis [39] as observed in pseudosuchians [40], tryonichids [37] and helochelydrids [41,42]. We then analysed the data with phylogenetic comparative methods (in Pseudosuchia and Testudinata) to reveal whether the post-cranial dermal bone vessel proliferation is: (1) influenced by the phylogeny and (2) correlated with lifestyle transitions unrelated to the phylogenetic relationships.

## 2. Material and methods

### (a) Sampling strategy

We studied 31 cross sections of dermal bones coming from different parts of the shell of both extant and extinct testudinatan species (dry bones and well-preserved fossils) from museum collections or published articles (table 1). The cross sections are transverse and pass by the centre of the sampled bones. The taxonomic affiliation and lifestyle attributes of the fossil forms could be identified unambiguously based on anatomical features. We classified the specimens into three categories depending on their lifestyle: terrestrial, freshwater and marine. When there was no ambiguity regarding the taphonomy (post-mortem transportation), the nature of the sediment was also used as a clue to infer their living environment (e.g. marine versus fresh water; electronic supplementary material, S1).

**Table 1.** Description of the sample, (*a*) Testudinata and (*b*) Pseudouchia. TMM: Texas Memorial Museum (Austin USA); FMNH: Field Museum of Natural History (Chicago, USA); MCNA: Museo de Ciencias Naturales de Alava (Vitoria-Gasteiz, Spain); YPM: Yale Peabody Museum (New Haven, USA); UPUAM: Unidad de Paleontología, Universidad Autónoma de Madrid (Spain); WU-SILS-RH: Waseda University (Tokyo, Japan); NSMT: National Museum for Nature and Science of Tokyo (Japan); ZIN PH: Zoological Institute (Russian Academy of Sciences, Saint Petersburg); FPDM: Fukui Prefectural Dinosaur Museum (Katsuyama City, Fukui Prefecture, Japan); UA: Université d'Antananarivo (Madagascar); SMNS: Smithsonian Institution; BSPG: Bayerische Staatssammlung für Paläontologie und Geologie, München, Germany; PEFO: Petrified Forest National Park, USA; ISI: Indian Statistical Institute (Calcutta, India); UCMP: University of California, Museum of Paleontology (Berkeley, USA); MNHN: Muséum National d'Histoire Naturelle; IPB: Institute of Paleontology (Bonn, Germany); NMS: Naturmuseum Solothurn, Switzerland; MCL: Musée des confluences (Lyon, France); PVL: Colección de Paleovertebrados del Instituto Miguel Lillo (Tucumán, Argentina); n.a.: non-attributed.

	porosity	lifestyle	region	ornamentation	age	collection number
(*a*) Testudinata						
*Hesperotestudo* sp.	0.07	terrestrial	flat osteoderm	no	Pleistocene	TMM 30967-1010.1
*Hesperotestudo* sp.	0.21	terrestrial	spiked osteoderm	no	Pleistocene	TMM 30967-1010.2
*Terrapene carolina tringuis*	0.40	terrestrial	neural	no	extant	FMNH 211806
*Terrapene carolina tringuis*	0.22	terrestrial	costal (right)	no	extant	FMNH 211806
*Dorkota vasconica*	0.21	freshwater	costal	no	Barremian	MCNA 14366
*Dorkota vasconica*	0.24	freshwater	neural	no	Barremian	MCNA 14372
*Podocnemis erythrocephala*	0.15	freshwater	sample costal	no	extant	YPM 11853
*Solemys* sp.	0.07	terrestrial	costal fragment	yes	Maastrichtyian	UPUAM-14001
*Solemys vermiculata*	0.14	terrestrial	costal fragment	yes	Maastrichtyian	MCNA 15047
*Solemys vermiculata*	0.16	terrestrial	shell fragment	yes	Maastrichtyian	MCNA 15046
*Carettochelys insculpta*	0.10	freshwater	costal (right 7th)	yes	extant	WU-SILS RH1044
*Pelodiscus sinensis*	0.11	freshwater	costal	yes	extant	NSMT-H 6600
Trionychidae indet.	0.11	freshwater	costal	yes	Aptian-Albian	ZIN PH 102
Trionychidae indet.	0.10	freshwater	costal	yes	early Cenomanian	ZIN PH 122
Trionychidae indet.	0.09	freshwater	costal	yes	Barremian–Aptian	FPDM V0127
*Bothremys barberi*	0.31	marine	costal	no	Campanian	FM P27406 (FMNH)
*Bothremys barberi*	0.32	marine	costal	no	Campanian	FM P27406 (FMNH)
*Bothremys barberi*	0.30	marine	neural	no	Campanian	FM P27406 (FMNH)
*Caretta caretta*	0.39	marine	costal	no	extant	FMNH 98963
*Caretta caretta*	0.33	marine	hyoplastron	no	extant	FMNH 98963
*Archelon ischyros*	0.26	marine	shell fragment	no	Late Cretaceous	YPM 1783
*Plesiochelys* sp.	0.18	marine	neural	no	Kimmeridgian	NMS 8730
*Taphrosphys sulcatus*	0.34	marine	costal	no	Maastrichtian	YPM 40288
*Taphrosphys sulcatus*	0.35	marine	neural	no	Maastrichtian	YPM 40288
*Ctenochelys stenoporus*	0.36	marine	neural	no	Campanian	FM PR 442
*Geochelone elegans*	0.14	terrestrial	costal	no	extant	IPB 561-C
*Geochelone elegans*	0.10	terrestrial	costal	no	extant	IPB 561-C
*Geochelone elegans*	0.07	terrestrial	neural	no	extant	IPB 561-C
*Hesperotestudo crassiscuta*	0.19	terrestrial	neural	no	Pleistocene	ROM 5540
*Hesperotestudo crassiscuta*	0.30	terrestrial	plaston fragment	no	Pleistocene	ROM 5541
*Hesperotestudo crassiscuta*	0.28	terrestrial	shell fragment	no	Pleistocene	ROM 5542
(*b*) Pseudosuchia						
*Araripesuchus tsangatsangana*	0.05	terrestrial	n.a.	yes	Late Cretaceous	UA 9966
*Batrachotomus kupferzellensis*	0.01	terrestrial	paramedian pre-caudal	yes	Late Ladinian	SMNS 80317
*Prestosuchus chiniquensis*	0.05	terrestrial	sacral paramedian	yes	Late Ladinian/Early Carnian	BSPG ASXXV7
*‘Prestosuchus’ loricatus*	0.04	terrestrial	pre-caudal paramedian	yes	Late Ladinian/Early Carnian	BSPG ASXXV46d
*Rauisuchus tiradentes*	0.16	terrestrial	pre-caudal paramedian	yes	Late Carnian/Early Norian	BSPG ASXXV121b
*Revueltosaurus* sp.	0.04	terrestrial	paramedian	yes	Norian	PEFO 33787
*Tikisuchus romeri*	0.14	terrestrial	pre-caudal paramedian	yes	Carnian	ISI R 305/ 1
*Simosuchus clarki*	0.1	terrestrial	n.a.	no	Late Cretaceous	UA 9965
*Simosuchus clarki*	0.07	terrestrial	n.a.	no	Late Cretaceous	UA 9965
*Yarasuchus deccanensis* (Avemetatarsalia)	0.10	terrestrial	pre-caudal paramedian	yes	Anisian	ISI R 334
*Alligator mississippiensis*	0.15	semi-aquatic	n.a.	yes	extant	SMNS 10481b
*Allognathosuchus wartheni*	0.13	semi-aquatic	n.a.	yes	Wasatchian	UCMP 113731
*Crocodylus niloticus*	0.13	semi-aquatic	dorsal	yes	extant	MNHN-AC- 1920.90, PC
*Diplocynodon* sp.	0.23	semi-aquatic	n.a.	yes	Eocene–Miocene	IPB R144 ⁄ 1
*Diplocynodon remensis*	0.24	semi-aquatic	nuchal	yes	Thanetian	MNHN. F. No number
*Machimosaurus hugii*	0.22	semi-aquatic	n.a.	yes	Late Jurassic	SMNS 81608
*Sarcosuchus imperator*	0.24	semi-aquatic	n.a.	yes	Upper Cretaceous	MNHN.F. GDF 380
*Steneosaurus* sp.	0.07	semi-aquatic	n.a.	yes	Late Jurassic	NMS 752
*Steneosaurus jugleri*	0.12	semi-aquatic	n.a.	yes	Late Jurassic	NMS 7152
*Paleosuchus trigonatus*	0.29	semi-aquatic	n.a.	yes	extant	MCL 420003939
*Protocaiman peligrensis*	0.13	semi-aquatic	n.a.	yes	Danian	UCMP 131693
*Teleosaurus cadomensis*	0.22	semi-aquatic	n.a.	yes	Bathonian	MNHN Histo 1960
*Goniopholis* sp.	0.2	semi-aquatic	n.a.	yes	Oxfordian-Berriasian	MNHN Histo 1727
*Borealosuchus* sp.	0.17	semi-aquatic	n.a	yes	Campanian-Ypresian	UCMP 133903
*Mahajangasuchus insignis*	0.17	semi-aquatic	n.a.	yes	Campanian	UA 9962
*Brachychampsa montana*	0.22	semi-aquatic	n.a.	yes	Maastrichtian	UCMP 133901
*Osteolaemus tetraspis*	0.12	semi-aquatic	n.a.	yes	extant	MNHN-AC-1991.4488
*Paleosuchus palpebrosus*	0.11	semi-aquatic	n.a.	yes	extant	MNHN.AC-1909.204
*Caiman crocodilus*	0.27	semi-aquatic	nuchal	yes	extant	Sorbonne Université - NA
*Borealosuchus wilsoni*	0.18	semi-aquatic	n.a.	yes	Ypresian	UCMP 131696
*Paratypothorax* sp.	0.25	terrestrial	paramedian	yes	Late Triassic	PEFO 5030
*Aetosaurus scagliai*	0.05	terrestrial	paramedian	yes	Late Triassic	PVL 2073

We sampled 32 cross sections of pseudosuchian osteoderms. The taxa were categorized based on two different lifestyles: terrestrial and semi-aquatic (table 1*b*). We decided not to distinguish the marine animals from the freshwater semi-aquatic forms as they have a similar amphibious ambush predator lifestyle [43,44]. The pelagic marine forms from the Jurassic (the metryorhinchids) [45,46] had completely lost the osteoderm shield and are therefore not suitable for this study (this aspect is discussed below). Extinct pseudosuchians are categorized based on the orientation of their skull neurosensory organs and limb postures as reviewed in a previous article [47].

### (b) Data acquisition

We produced photographs of each cross section before segmenting and rendering them binary in black and white with Adobe Photoshop CC 2015 (electronic supplementary material, S2 and S3), so that the bone matrix appears in black and the empty cavities appear in white. The ornamentation of dermal bones is often made of crests separated by pits, which systematically host large clusters of blood vessels from the soft dermis. These blood vessels are intimately associated with the dermal bone vascularization as they pierce its surface [39] and connect vessel clusters in the internal cavities (spongiosa) via these vascular openings at the surface of the bone [37,39]. As pseudosuchians [48], tryonichids [37] and helochelydrids [41] possess ornamented post-cranial dermal bones whose dermis vascularization is directly related to the dermal bone internal vascularization. Although these pits are large, they obviously do not over-estimate the porosity of the bone as they are fully filled in by a great number of blood vessels [39]. For that reason, we decided to include the space of these pits into our measurements. To do so, we connected the top of the crests of the ornamentation with a virtual black line of one pixel in the most parsimonious way to embed the surface of the pits into the vascular measurements (electronic supplementary material, S3). We exported the pictures in TIFF format (electronic supplementary material, S2) and analysed them with Bone profiler [49] in order to measure the area occupied by the empty spaces proportionally to the entire area covered by bone and vascular spaces (as detailed in electronic supplementary material, S3).

### (c) Phylogenetic comparative analyses

**Figure 1.**
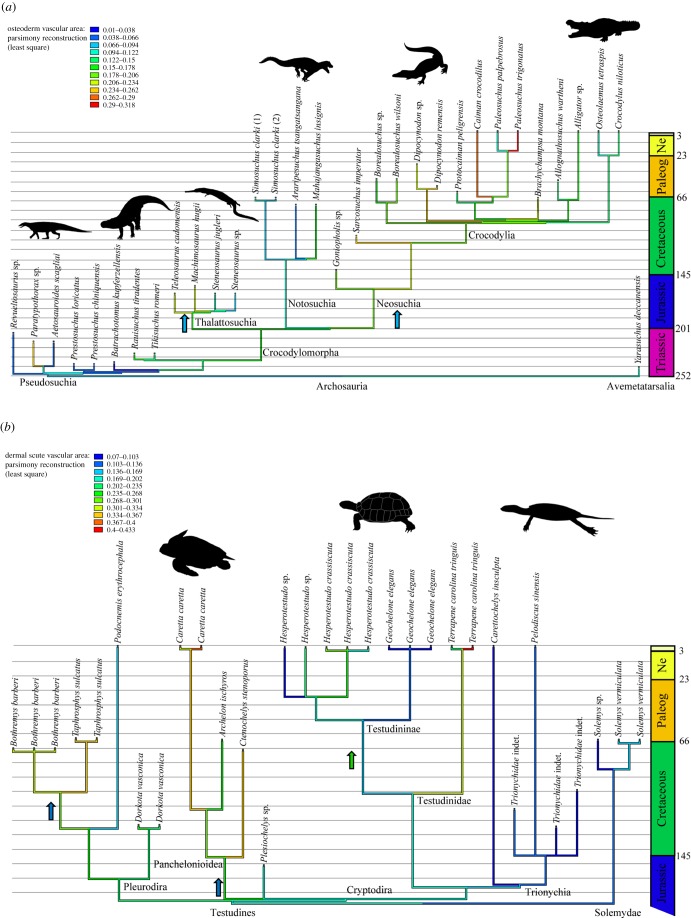
(*a*) Reconstruction of the osteoderm vascular area on the phylogeny of Pseudosuchia using a least-square reconstruction. The phylogeny was reconstructed and time-scaled according to published references [5,51–58]. The light blue arrows represent the transitions from a terrestrial to a semi-aquatic lifestyle. (*b*) Reconstruction of the dermal scute vascular area on the phylogeny of Testudinata using a least-square reconstruction. The phylogeny was reconstructed and time-scaled according to published references [41,42,59–63]. The dark blue arrows represent the transitions from a freshwater to a marine lifestyle. The green arrow represents a transition from a freshwater to a terrestrial lifestyle. Regarding the dermal plates, which belong to the same species or specimen, we decided to separate them from a 1 Myr-old last hypothetical common ancestor: a systematic error which is below 1% when considering the total branch length within the phylogeny timescale (180 Ma for the testudinatans and 250 Ma for the pseudosuchians). Paleog, Paleogene, Ne, Neogene.

For both Pseudosuchia and Testudinata, time-scaled phylogenetic relationships of the sampled specimens were reconstructed in Mesquite [50] by relying on published references [5,41,42,51–63] (figure 1). We then traced the evolution of the post-cranial dermal bone vascular area using the least-squared parsimony to calculate the ancestral states for each clade. In order to test the influence of the phylogeny (i.e. the historical constraint) on the vascular area of the post-cranial dermal skeleton, we exported the trees in NEX format into R [64] and we further computed both Pagel's *λ* [65] and Blomberg's *K* [66] after uploading the ‘caper’ package [67,68]. Finally, we tested the correlation between the post-cranial dermal bone vascular area and the corresponding lifestyle for each taxon using a phylogenetic ANOVA. This is a statistical test that reveals a correlation between quantitative and qualitative data while retracting the influence of the phylogeny, which is quantified either by *K* or *λ*—we decided to consider both options [68] (table 2).

**Table 2.** Statistical results. s.d.: standard deviation; max: maximum value; min: minimum value.

Pseudosuchia			Testudinata		
phylogenetic analyses	result/value	*p*-value	phylogenetic analyses	result/value	*p*-value
*K*	significant/*K* = 0.41	0.004	*K*	significant/*K* = 0.09	0.003
ANOVA(*K*)	non-significant	0.21	ANOVA(*K*)	non-significant	0.6658
*λ*	significant/*λ* = 0.99	1.97 × 10^−5^	*λ*	significant/*λ* = 0.83	2.48 × 10^−5^
ANOVA(*λ*)	non-significant	0.21	ANOVA(*λ*)	non-significant	0.6658

statistical results			statistical results		
lifestyle	terrestrial	semi-aquatic	terrestrial	freshwater	marine
mean porosity	0.09	0.18	0.18	0.14	0.31
median	0.06	0.19	0.16	0.11	0.33
s.d.	0.07	0.06	0.10	0.06	0.06
min	0.01	0.07	0.07	0.09	0.18
max	0.25	0.29	0.40	0.24	0.39

## 3. Results

### (a) Evolution of vascular density in the osteoderms of Pseudosuchia

We first tested whether the variability of the vascular area—proportionally to the dermal bone area—was inherited from the phylogenetic relationships of the studied species. Phylogenetic tests showed that the vascular area in the osteoderms of the pseudosuchians is significantly influenced by the phylogeny, as both the Blomberg's *K* and the Pagel's *λ* tests are significant (*p*-values of less than 0.05; table 2). The fact that the *λ*-value of 0.99 is very close to the maximum (*λ* = 1) means that the vascular area covaries in direct proportion with the species' shared evolutionary history through a Brownian motion on the phylogeny [65,69]. The *K*-test shows a significant *p*-value (table 2) and thus emphasizes the tendency of closely related species to share a similar osteoderm vascular area. Nevertheless, as the *K*-value itself clearly remains below 1, the phylogeny must not be the only component that explains the resulting evolutionary pattern of vascular area variability within Pseudosuchia [66,70].

**Figure 2.**
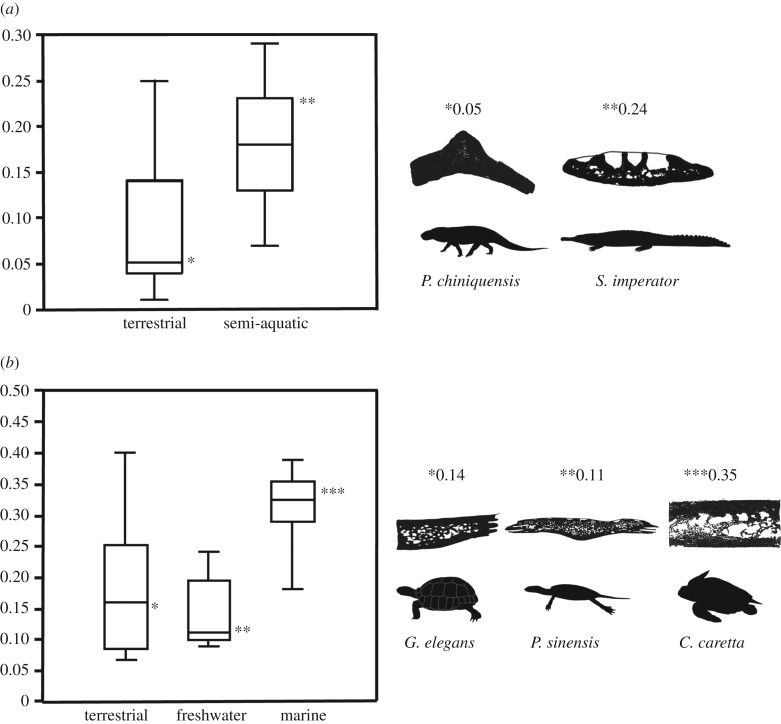
(*a*) Boxplot of the osteoderm vascular area in the pseudosuchians. (*b*) Boxplot of the dermal scute vascular area in the testudinatans. The four quartiles represent the dispersion of the values for each lifestyle.

A first glimpse of the vascular density distribution would suggest that the lifestyle of the studied taxa could partly explain this variability. Boxplots were calculated to illustrate the distribution of the vascular cross-sectional area in the osteoderms of semi-aquatic and terrestrial pseudosuchians. Semi-aquatic forms exhibit a larger vascular area (proportionally to their dermal bone area) than terrestrial pseudosuchians (while showing a more pronounced apical ornamentation; table 2 and figure 2*a*; electronic supplementary material, S2). Indeed, although both datasets show an equal standard deviation (s.d._terrestrial_ = 0.07; s.d._semi-aquatic_ = 0.06), the mean value of the osteoderm vascular area is equal to 0.09 in the terrestrial forms, whereas it is twice as high in the semi-aquatic pseudosuchians (mean = 0.18).

In order to test whether this distribution could partly explain the variability of vascular density in Pseudosuchia, we performed a phylogenetic ANOVA that takes into account the phylogenetic signal. The results present no significant correlation between the osteoderm vascular area and the pseudosuchian lifestyle (table 2).

### (b) Evolution of vascular density in the shell of Testudinata

We evaluated the vascular areas of turtle shell in a phylogenetic context. They show that the dermal shell bone vascular density is significantly influenced by the phylogeny since both the Blomberg's *K* and the Pagel's *λ* tests are significant (*p*-values of less than 0.05; table 2). However, both the *λ* and the *K* show lower values (*λ* = 0.83; *K* = 0.09) than for the pseudosuchian osteoderm vascular area (*λ* = 0.99; *K* = 0.41). We deduce that the phylogeny explains to a lesser extent the variability of shell vascular density in testudinatans than in pseudosuchians (figure 1*b*).

As presented with boxplots (figure 2*b*) and in table 2, the testudinatan dermal bone vascular area seems to score higher values than in the pseudosuchian osteoderms although the standard deviation is equal, with the exception of terrestrial testudinatans, whose vascular density varies in a larger spectrum around a mean value of 0.18 (s.d. = 0.10). Unlike semi-aquatic pseudosuchians (which are most often found in freshwater environments), the freshwater turtle dermal bones show a lower vascular density (mean = 0.14) than terrestrial forms (mean = 0.18). The presence of ornamentation in both Trionychia and in Helochelydridae does not seem to influence the global turtle dermal bone vascularity as these bones still score a low vascular area (all values are lower than 0.16; table 2; see Material and methods). As a third category, the marine turtles, which are fully aquatic (with a very brief terrestrial excursion on land for females to lay eggs), present a high average value of shell vascular area (mean = 0.31) with a standard deviation similar to that of the freshwater turtles (figure 2*b* and table 2).

A phylogenetic ANOVA was performed and shows that the vascular area in the testudinatan post-cranial dermal bones was not significantly correlated with their lifestyle (table 2), despite these discrete boxplot distributions.

## 4. Discussion

### (a) Pseudosuchian osteoderm vascularization: historical constraints versus ecophysiological adaptations

Our results show that the variability of the osteoderm vascularization correlates with the phylogenetic relationships within Pseudosuchia. Although the lifestyle seems to partly explain the rest of the correlation factor according to the global distribution of the data, our phylogenetic ANOVA revealed no significant correlation between osteoderm vascular variability and lifestyle. The high osteoderm vascularity in the semi-aquatic forms was therefore likely the result of a historical constraint (as evidenced by the significant values of *λ* and *K*) rather than an ecological adaptation based on natural selection. Nevertheless, some recent studies on living species have shown that the bone cavities in the crocodylian osteoderms reveal an enclosed vascular proliferation [39], which is involved in acidosis buffering during prolonged apnea [30], as well as in heat transfer during emerged and semi-emerged basking periods [23]. Therefore, we cannot refute the existence of such physiological mechanisms in the extinct crocodylomorphs who shared the same semi-aquatic ambush predator behaviour as the extant crocodylians: the extinct neosuchians (e.g. *Sarcosuchus imperator*, *Goniopholis* sp*.*) [31,71,72] and the teleosaurids [45,55,73].

Regarding the thalattosuchians that adopted a pelagic lifestyle involving long-term apneas (Metriorhynchidae; [44–46]), the loss of the dermal shield must have negatively impacted their performance in bone acidosis buffering. Nevertheless, other pathways can buffer acidosis via the involvement of soft tissues [74]. Such mechanisms have already been observed in marine birds and mammals [75–77]. Contrary to extant crocodylians, marine birds and mammals are very active swimmers. Their lack of oxygen due to apnea essentially affects their appendicular musculature. To compensate for the acidity increase, limb muscles synthetize a protein (carnosine) [78–80] which complexes with protons and thus buffers the intracellular acidosis in muscle tissues where free oxygen concentration is the lowest. As the fossil forms—metriorhynchids—probably were active sea predators, as evidenced by the presence of a tail fluke and swimming paddles [45,46], we can assume that metabolic acidosis buffering could have involved the muscular system as in extant marine birds and mammals. However, the reasons for the metriorhynchids to have lost their osteoderm shield remain unknown. This loss could reflect a complex conjuncture involving both phylogenetic and structural constraints influencing the development of the dorsal shield in disregard of its physiological implication(s) (i.e. weight loss, flexibility along the anteroposterior axis, etc. [45,46,81]).

### (b) Testudinatan shell vascularization: historical constraints versus ecophysiological adaptations

Likewise, our results show that the testudinatan shell vascular density is essentially constrained by the phylogeny despite the noticeable differences in the mean values of vascular area between taxa belonging to different lifestyle categories (table 2 and figure 2*b*).

Higher porosity is encountered in the marine forms. It probably provides a dense vascular system, which facilitates long-term apnea via bone acidosis buffering since this function is essential to sea turtles, of which only the females emerge on land for nesting [82]. Most of their feeding habits rely on a vegetarian or omnivorous diet from the sea bottom [83]. Density reduction due to the lightening of the shell bone perforated by a large number of vascular canals obviously increases their buoyancy and intensifies their effort to dive and remain at the bottom of the sea. Because the control of buoyancy is moderated by the lungs [84,85], we strongly suspect that the porosity of the shell could be better explained as the result of physiological functions such as bone acidosis buffering than in relation to biomechanics.

Unlike the marine forms, the freshwater turtles do not exhibit a high shell bone porosity although they are known to perform bone acidosis buffering during prolonged apnea and while hibernating in freezing and/or anoxic conditions [24–30,86]. Some freshwater species such as the trionychids seem to have developed a different strategy to withstand long duration apneas. Indeed, in comparison with the other freshwater testudinatans, the trionychids are known to have a lower performance in bone acidosis buffering as they are less tolerant to anoxia [87]. Instead, they exchange blood gases with those dissolved in the surrounding water using pharyngeal, cloacal and cutaneous respiration [88]. Although gas exchanges are not possible through scales of keratin in sauropsids (including crocodylians and testudinatans), this mechanism is rendered possible in trionychids by the secondary loss of their superficial keratin layer [63]. It is worth mentioning that the use of cutaneous respiration in Trionychia correlates with a rare expression of shell apical ornamentation in testudines. As illustrated in previous studies [37], the pits which compose the trionychid shell sculpture always house one or several vascular openings which provide a proliferation of superficial vessels as in crocodylian ornamented osteoderms [39]. This configuration provides a large blood-vessel network for gas exchanges in cutaneous respiration [89].

Even if heat exchange with the environment is vital for testudinatans [90], which are ectotherms, this function does not seem to correlate with the evolutionary pattern of shield vascularization (considering the vascular area as a proxy). Indeed, both freshwater and terrestrial turtle dermal bones globally show a lower vascular density than marine forms, although temperature variations in the sea are much narrower than on land or in freshwater environments.

In conclusion, the dermal shield of the testudinatans seems to play multiple physiological roles which differently concern: (1) marine turtles (acidosis buffering during prolonged apnea); (2) freshwater turtles (cutaneous respiration and/or acidosis buffering in response to prolonged apnea or hibernation in anoxic or freezing conditions, heat transfer when basking); and (3) terrestrial tortoises (heat transfer). Therefore, it seems unlikely that any resulting combination of these functions represents the primary determinant of morphology once we have considered the influence of the phylogenetic relationships (historical constraints).

### (c) Evolutionary trends in Pseudosuchia and Testudinata

Pseudosuchians and testudinatans have been repeatedly defined as sister taxa according to several phylogenetic reconstructions [91–97]. Among amniotes, these two groups are the main ones to have developed a large post-cranial dermal skeleton which is known to be used in both acidosis buffering and heat transfer. This pattern leads us to wonder if this ability of the post-cranial dermal bones to perform ecophysiological functions results from a phylogenetic heritage or consists of a functional analogy. The vascular density in the post-cranial dermal skeleton increased when pseudosuchians transited to a semi-aquatic lifestyle in the Early Jurassic and when turtles transited to a marine lifestyle (within Pleurodira during the Cretaceous, within Cryptodira during the Jurassic and maybe in some early testudinatan species such as *Eileanchelys waldmani* and *Heckerochelys romani* for which the assumed marine lifestyle is still debated [38,98]; figures 1 and 2). All these transitions probably induced bone acidosis buffering to balance prolonged apnea, which directly depends on the bone vascularization inside the shell cavities [29].

Even though the vascularization in post-cranial dermal bones must also be involved in heat transfer due to its peripheral location on the body [20,35], testudinatans and pseudosuchians nevertheless have evolved through very different thermal metabolism patterns. Indeed, the pseudosuchians derive from an endothermic ancestor [99–103] whereas ectothermy is a plesiomorphic condition in Testudinata [38,104]. The increase in dermal bone vascularization relates to heat transfer in the semi-aquatic crocodylomorphs [23,39,47] but this process does not explain the observed pattern in the testudinatan shell as the terrestrial forms score lower relative vascular area although they are the most exposed to external thermal variations.

The functional role(s) of bone ornamentation may also differ between the turtles and the crocodylians. Indeed, even though the vascular openings within the ornamental pits must play a role in cutaneous respiration in soft-shell turtles, this function is not possible in the crocodylians since their entire body is covered by a layer of keratine [31]. The function(s) of bone ornamentation in Crocodylomorpha must instead concern acidosis buffering and heat transfer via the housing of vessel clusters straight over the bone apical surface in connection with the blood vessels underneath, which are enclosed in the bone cavities within the osteoderm core (spongiosa).

As a conclusion, we suggest that the vascular plasticity of the post-cranial dermal bones in both Testudinata and Pseudosuchia probably helped these clades make major evolutionary shifts by offering various pathways to oxygen and/or heat management. Despite the fact that upshifts in vascular density often relate to an increased frequency of internal low oxygen due to a freshwater or marine lifestyle, we do not exclude that vascular density also relates to other vital functions as well as historical and structural constraints which drive the development and morphology of the dermal plates [105]. The complexity of multi-functional roles of the post-cranial dermal skeleton in both pseudosuchians and testudinatans might be a reason why our phylogenetic ANOVA revealed no relation between ‘vascular area’ and ‘ecology’ despite obvious differences between the lifestyle categories. Our results however demonstrate that the advanced development of a post-cranial skeleton in these groups was crucial for the survival and dispersal of these taxa in various ecological niches. This major evolutionary step should be more thoroughly investigated.

## Supplementary Material

The lifestyle of the extinct testudinians

## Supplementary Material

Image dataset

## Supplementary Material

Detailed protocol

## Data accessibility

The data are accessible in electronic supplementary material files.
