# Supplementary material for: The evolution of dermal shield vascularization in Testudinata and Pseudosuchia: phylogenetic constraints versus ecophysiological adaptations
Source: Philos Trans R Soc Lond B Biol Sci. 2020 Jan 13;375(1793):20190132. doi: 10.1098/rstb.2019.0132 (PMC7017437; doi:10.1098/rstb.2019.0132)
Supplement: The lifestyle of the extinct testudinians [file rstb20190132supp1.docx]

| **Taxa** | **Lifestyle** | **Anatomical and ecological justification** | **References** |
| --- | --- | --- | --- |
| *Hesperotestudo* sp*.* | Terrestrial | - very high-domed shell  - stout limbs  - skull with triturating surfaces indicating herbivory  - presence of osteoderms on the tail and limbs | Meyland and Sterrer  2000 |
| *Dorkota vasconica* | Freshwater | presence of large fontanelles which remain open throughout ontogeny | Pérez-García et al. 2012 |
| *Solemys* sp. | Terrestrial | presence of limb osteoderms | Joyce et al.  2011 |
| Trionychidae *indet.* | Freshwater | presence of a flat, flexible, and de-scaled shell | Nakajima et al. 2017 |
| *Bothremys barberi* | Marine | - found in marine sediments  - forelimb morphology indicating a shallow sea bottom walker | Gaffney et al.  2006  Hirayama and Hoshida  2018 |
| *Archelon ischyros* | Marine | giant turtle with digits elongated in flippers | Wieland 1896  Williston 1914 |
| *Plesiochelys* sp*.* | Marine | - large foramina interorbitale for accommodation of hypertrophied salt glands in the skull  - forelimbs modified into paddles  - found in marine sediments | Anquetin et al.  2014 |
| *Taphrosphys sulcatus* | Marine | found in marine sediments | Lapparent and Werner 1998 |
| *Ctenochelys stenoporus* | Marine | - carapace with large lateral fontanelles between costals as in modern sea turtles  - found in marine sediments | Karl and Nyhuis  2012 |
| *Hesperotestudo crassiscuta* | Terrestrial | - very high-domed shell  - stout limbs  - skull with triturating surfaces indicating herbivory  - presence of osteoderms on the tail and limbs | Meylan and Sterrer 2000 |

Supplementary file 1: Category affiliation of the sampled extinct testudinatans.

References :

Meylan PA, Sterrer W. 2000. *Hesperotestudo* (Testudines: Testudinidae) from the Pleistocene of Bermuda, with comments on the phylogenetic position of the genus. *Zool. J. linn. Soc.* **128**, 51-76. (doi: 10.1006/zjls.1998.0199)

Perez-Garcia A, Scheyer TM, Murelaga X. 2012. New interpretations of *Dortoka vasconica* Lapparent de Broin and Murelaga, a freshwater turtle with an unusual carapace. *Cretac. Res.* **36**, 151-161. (doi: 10.1016/j.crestres.2012.03.006)

Joyce WG, Chapman SD, Moody RTJ, Walker CA. 2011. The skull of the solemydid turtle *Helochelydra nopcsai* from the early cretaceous of the isle of Wight (UK) and a review of Solemydidae. *Palaeontology* **86**, 75-97. (doi: 10.1111/j.1475-4983.2011.01075.x)

Nakajima Y, Danilov IG, Hirayama R, Sonoda T, Scheyer TM. 2017. Morphological and histological evidence for the oldest known softshell turtles from Japan. *J. Vertebr. Paleontol.* **37(2)**, e1278606. (doi:10.1080/02724634.2017.1278606)

Gaffney ES, Tong H, Meylan PA. 2006. Evolution of the side-necked turtles: the families Bothremydidae, Euraxemydidae, and Araripemydidae. *B. Am. Mus. Nat. Hist.* **300**, 1-698. (doi: /10.1206/0003-0090)

Hirayama R, Yoshida M. 2018. Limb morphology of the genus *Bothremys* (Bothremydidae;

Pleurodira) from the Late Cretaceous of Morocco, Northern Africa. In *the Turtle Evolution Symposium* (eds R Hirayama and the Host Committee of the 6th Turtle Evolution Symposium), 55. Scidinge Hall Verlag Tübingen, ISBN 978-3-947020-06-5. Tokyo, Japan

Wieland GR. 1896. *Archelon ischyros*: a new gigantic cryptodire testudinate from the Fort Pierre Cretaceous of South Dakota. *Am. J. Sci.* **4(2)**, 399-412.

Williston SW. 1914. *Chelonia. Water Reptiles of the Past and Present*. pp. 231-241. Chicago, USA: University of Chicago Press.

Cadena EA, Parham JF. 2015. Oldest known marine turtle? A new protostegid from the Lower Cretaceous of Colombia. *PaleoBios* **32**, 1–42.

Anquetin J, Deschamps S, Claude J. 2014. The rediscovery and redescription of the holotype of the Jurassic turtle *Plesiochelys etalloni*. *Peer J2*: e258. (doi: 10.7717/peerj.258)

Lapparent de Broin F, Werner C. 1998. New late Cretaceous turtles from the Western Desert, Egypt. *Ann. Paleontol.* **84(2)**, 131-214.

Karl HV, Nyhuis CJ. 2012. *Ctenochelys stenoporus* (Hay, 1905) (Testudines : Toxochelyidae) and *Clidastes* sp. (Squamata : Mosasauridae) from the upper Cretaceous of NW-Germany. *Studia Palaeocheloniologica* **4(9)**, 129-142.
