# Supplementary figures and images for: The evolution of dermal shield vascularization in Testudinata and Pseudosuchia: phylogenetic constraints versus ecophysiological adaptations
Source: Philos Trans R Soc Lond B Biol Sci. 2020 Jan 13;375(1793):20190132. doi: 10.1098/rstb.2019.0132 (PMC7017437; doi:10.1098/rstb.2019.0132)

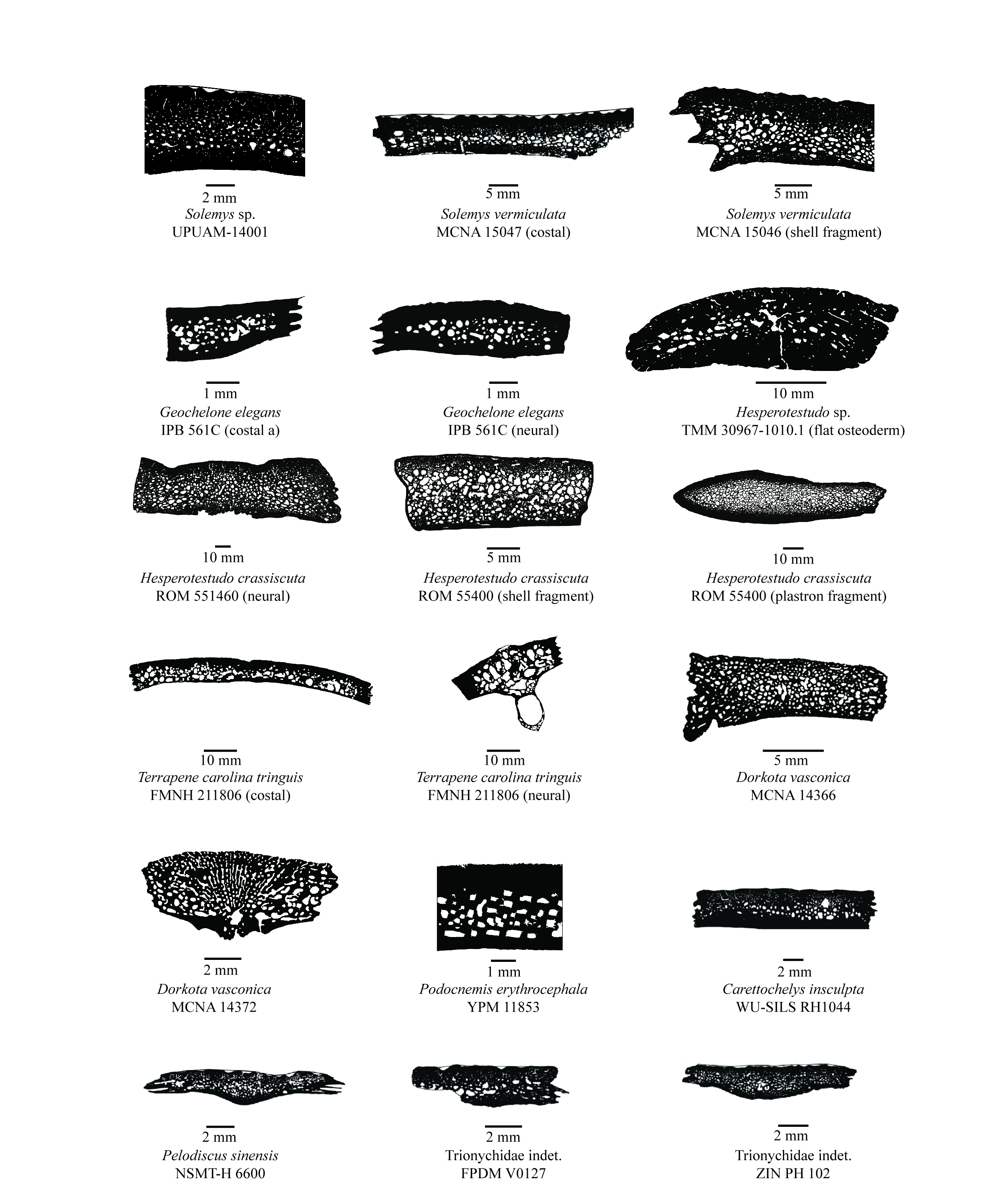

Supplement: Image dataset [file rstb20190132supp2.zip › rstb20190132_si_004.tif]

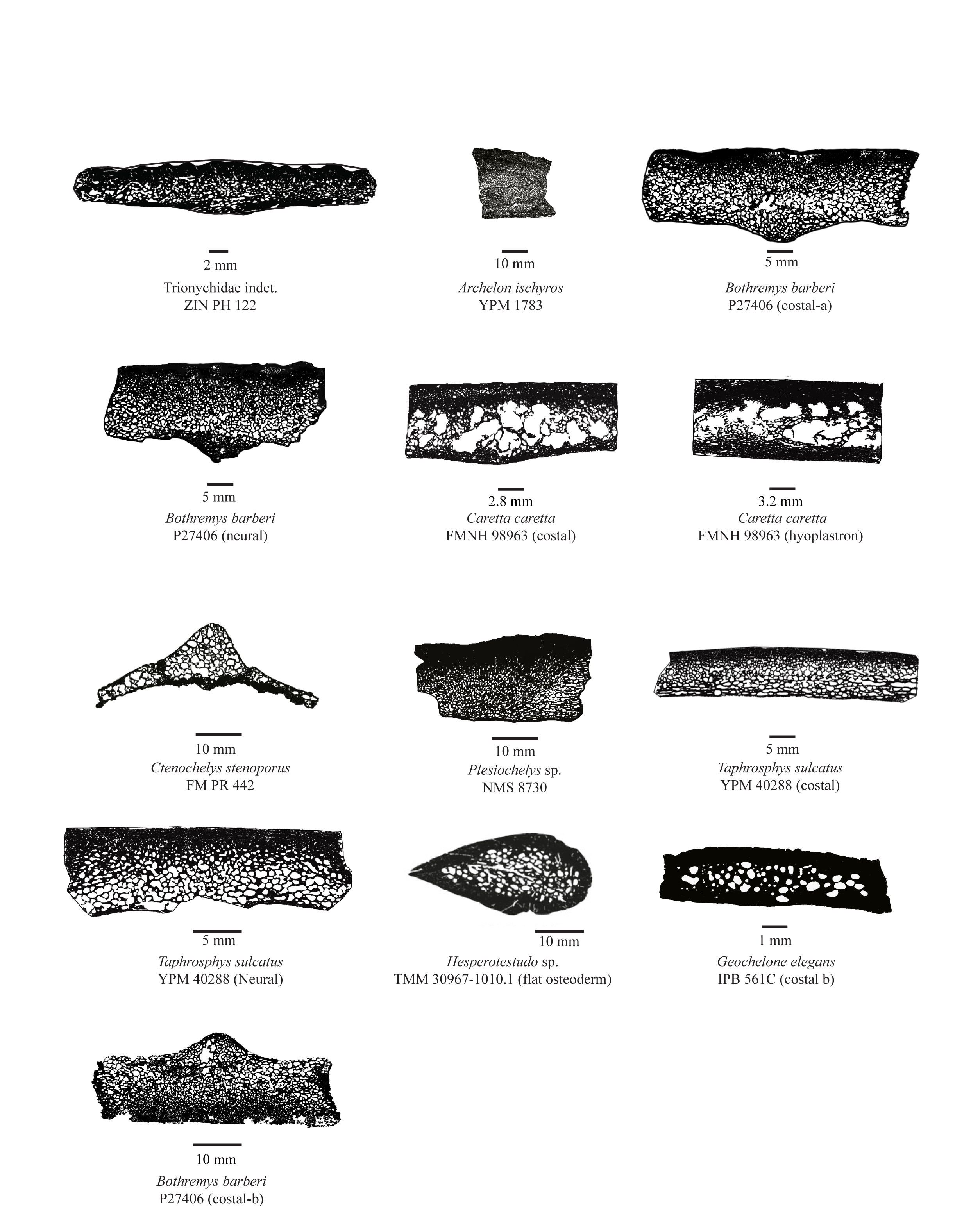

Supplement: Image dataset [file rstb20190132supp2.zip › rstb20190132_si_005.tif]

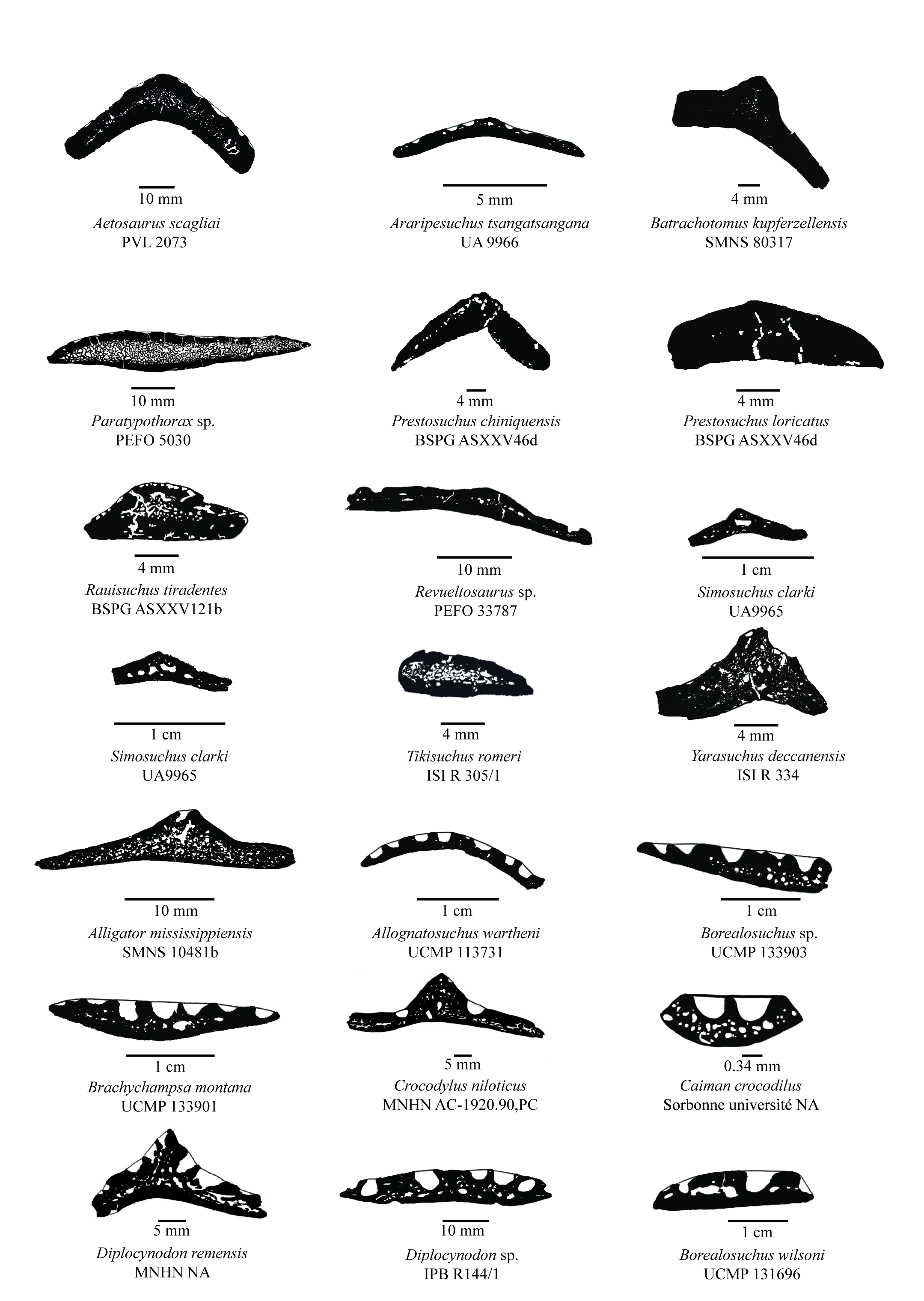

Supplement: Image dataset [file rstb20190132supp2.zip › rstb20190132_si_002.tif]

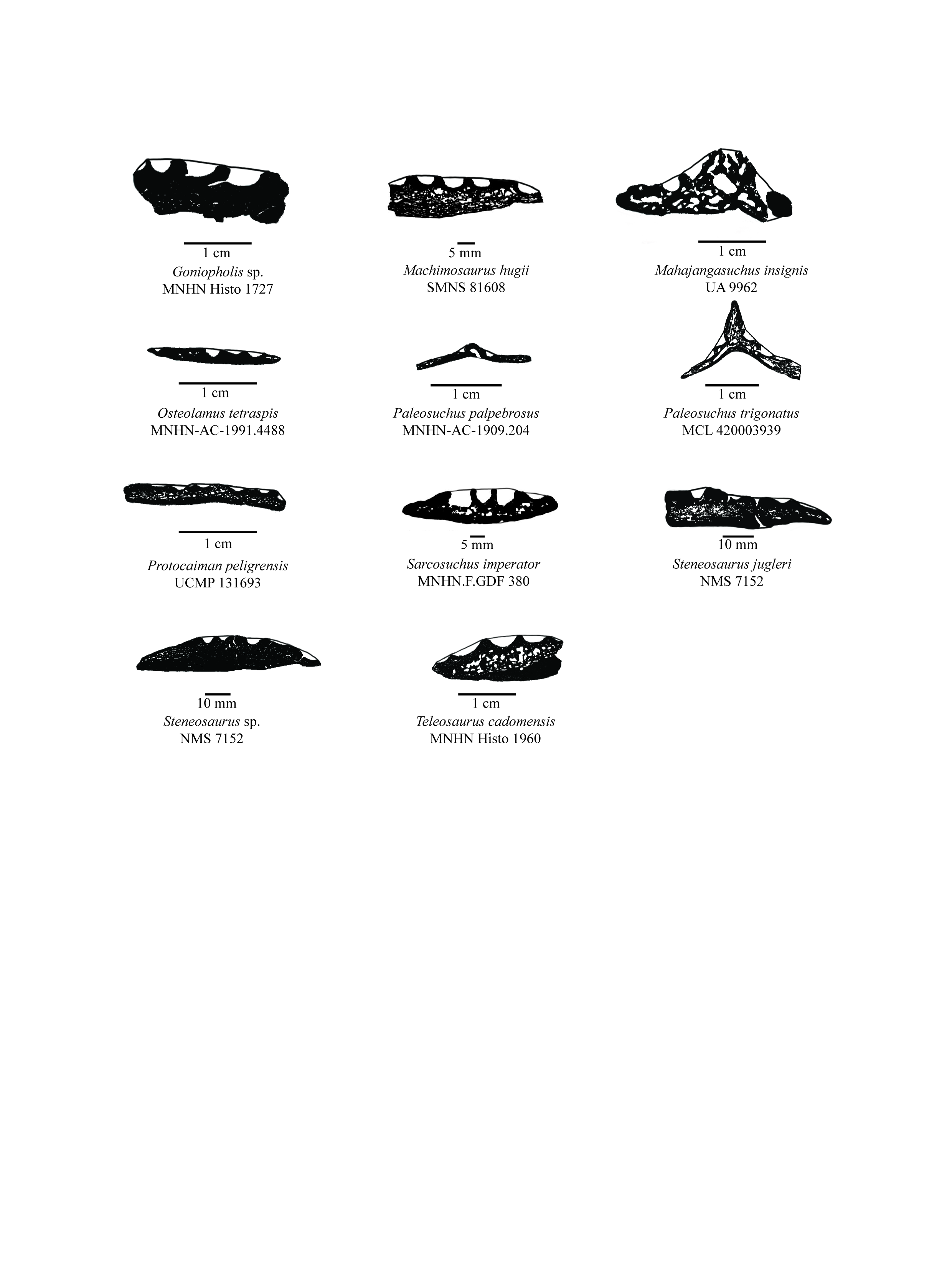

Supplement: Image dataset [file rstb20190132supp2.zip › rstb20190132_si_003.tif]
